# Development of novel InDel markers and genetic diversity in *Chenopodium quinoa* through whole-genome re-sequencing

**DOI:** 10.1186/s12864-017-4093-8

**Published:** 2017-09-05

**Authors:** Tifu Zhang, Minfeng Gu, Yuhe Liu, Yuanda Lv, Ling Zhou, Haiyan Lu, Shuaiqiang Liang, Huabin Bao, Han Zhao

**Affiliations:** 1 Provincial Key Laboratory of Agrobiology, Institute of Crop Germplasm and Biotechnology, Jiangsu Academy of Agricultural Sciences, Nanjing, Jiangsu 210014 China; 2 Xinyang Agricultural Experiment Station of Yancheng City, Yancheng, Jiangsu 224336 China; 3 Department of Crop Sciences, University of Illinois at Urbana-Champaign, Urbana, IL 61801 USA

**Keywords:** Quinoa, SNP, InDel, Genetic diversity, Population structure, Core germplasm

## Abstract

**Background:**

Quinoa (*Chenopodium quinoa* Willd.) is a balanced nutritional crop, but its breeding improvement has been limited by the lack of information on its genetics and genomics. Therefore, it is necessary to obtain knowledge on genomic variation, population structure, and genetic diversity and to develop novel Insertion/Deletion (InDel) markers for quinoa by whole-genome re-sequencing.

**Results:**

We re-sequenced 11 quinoa accessions and obtained a coverage depth between approximately 7× to 23× the quinoa genome. Based on the 1453-megabase (Mb) assembly from the reference accession Riobamba, 8,441,022 filtered bi-allelic single nucleotide polymorphisms (SNPs) and 842,783 filtered InDels were identified, with an estimated SNP and InDel density of 5.81 and 0.58 per kilobase (kb). From the genomic InDel variations, 85 dimorphic InDel markers were newly developed and validated. Together with the 62 simple sequence repeat (SSR) markers reported, a total of 147 markers were used for genotyping the 129 quinoa accessions. Molecular grouping analysis showed classification into two major groups, the Andean highland (composed of the northern and southern highland subgroups) and Chilean coastal, based on combined STRUCTURE, phylogenetic tree and PCA (Principle Component Analysis) analyses. Further analysis of the genetic diversity exhibited a decreasing tendency from the Chilean coast group to the Andean highland group, and the gene flow between subgroups was more frequent than that between the two subgroups and the Chilean coastal group. The majority of the variations (approximately 70%) were found through an analysis of molecular variation (AMOVA) due to the diversity between the groups. This was congruent with the observation of a highly significant F_ST_ value (0.705) between the groups, demonstrating significant genetic differentiation between the Andean highland type of quinoa and the Chilean coastal type. Moreover, a core set of 16 quinoa germplasms that capture all 362 alleles was selected using a simulated annealing method.

**Conclusions:**

The large number of SNPs and InDels identified in this study demonstrated that the quinoa genome is enriched with genomic variations. Genetic population structure, genetic core germplasms and dimorphic InDel markers are useful resources for genetic analysis and quinoa breeding.

**Electronic supplementary material:**

The online version of this article (10.1186/s12864-017-4093-8) contains supplementary material, which is available to authorized users.

## Background

Quinoa (*Chenopodium quinoa* Willd.) is an important seed crop native to the Andean region of South America and has been widely cultivated in Bolivia, Peru and Chile. The earliest quinoa domestication period can be traced back to 5000 BC in Chile [1]. As a major protein source, quinoa has played a crucial role in stable food supplies and nutritional supplements for local civilizations. Currently, the market demand for quinoa as a “superfood” has expanded to North America, Europe and Japan due to its unique nutritional characteristics, including a balanced amino acid profile and lack of gluten in its seeds [2, 3]. Additionally, quinoa has great abiotic tolerance to salt and drought. These features have attracted researchers to better understand the underlying genetics and genomics of quinoa [4–13].

Quinoa is an allotetraploid species (2*n* = 4*×* = 36). Its genome size is estimated to be 1448 megabases (Mb) [14–17], placing it in between two major diploid crops: rice (430 Mb) and maize (2500 Mb) [18, 19]. Because quinoa has two distinct subgenomes, A and B, its genome is more complex than a normal diploid species [20]. Two primary versions of the assembled quinoa genome based on two different varieties have been recently released (Cqu_r1.0 http://quinoa.kazusa.or.jp/ and *Chenopodium quinoa v1.0* http://phytozome.jgi.doe.gov/) [15, 21]. Compared with Cqu_r1.0, *Chenopodium quinoa v1.0* represents a high-quality, chromosome-scale reference genome of quinoa with 44,776 annotated gene models [21]. However, genomic variations in quinoa, such as single nucleotide polymorphisms (SNPs) and Insertions/Deletions (InDels), have not been comprehensively characterized. Therefore, re-sequencing the diverse quinoa germplasm genomes is necessary to obtain a better understanding of genomic variation within the species.

Some types of molecular markers, such as random amplified polymorphic DNA (RAPD), amplified fragment length polymorphism (AFLP), simple sequence repeat (SSR), and SNP, have been identified in quinoa [1, 22–28]. According to its morphological, distributional and agronomic criteria, five groups of quinoa were first reported, including the Valle, Altiplano, Yungas and Salares groups in the highlands of South America and the Nivel del Mar group along the south-central coast of Chile [29]. In a subsequent study, the quinoa groups were classified into the following two main types using 21 isozyme loci and two morphological traits: (1) the coastal type from southwestern Chile and (2) the Andean highland type from northwestern Argentina to southern Colombia; the highland type was further subdivided into the northern and southern subgroups [30]. These grouping efforts were supported by the clusters of 143 accessions from the United States Department of Agriculture (USDA) based on 36 highly reproducible SSRs [31]. Two other similar studies were conducted on regional quinoas using fewer SSRs [32, 33]. Additionally, a genetic study using 427 SNPs from 113 USDA quinoa accessions further supported the aforementioned grouping results [24, 30, 31].

In genetic diversity studies, a core set of germplasms has been used to best capture the number of allelic variations and represents the genetic diversity with only a small number of individuals. Core sets have been reported for several crop species such as maize, rice and cotton using SSR markers [34–36]. In quinoa, however, the only two reports on core collections were limited to Peru, and both were only based on geographical or morphological information [37, 38]. The selection of a core set representing major quinoa planting areas in South America by molecular marker analysis has not been reported.

Until now, genetic analysis of quinoa was mainly conducted using a limited number of SSR markers screened from single source [25, 27]. Due to the allotetraploidy nature of quinoa, some SSR markers could produce four or more amplicons, which makes genotyping results hard to interpret and record. Thus, dimorphic molecular markers would be the better choice for genotyping a polyploidy species such as quinoa. In this study, the main objectives were to (1) characterize the SNPs and InDel variations in the quinoa genome via de novo re-sequencing; (2) develop new dimorphic InDel markers genome-wide; (3) analyze population structure and genetic diversity among quinoa accessions; and (4) select a quinoa core set that is representative of the main planting areas.

## Methods

### Plant germplasm

In total, 129 quinoa accessions were collected for analysis, including 123 accessions provided by the United States Department of Agriculture-National Plant Germplasm System (USDA-NPGS) and six private accessions (Table 1). These quinoa accessions primarily represent the germplasms from South and North America. Of these, 42 accessions from the USDA-NPGS were donated by Emigdio Ballón where the assigned origin place of “United States, New Mexico” was actually inaccurate [31]. The likely origin of the six private accessions was not clear, and their collection place was used in its place.

**Table 1:** The list of quinoa from USDA and private collection

Serial number	Accesion	Plant name	Origin	Source
1	Ames 13,214	Chucapaca	Bolivia, La Paz	USDA-NPGS
2^a^	Ames 13,228	DE-1	Ecuador, Otavalo	USDA-NPGS
3	Ames 13,719	27 GR	United States, New Mexico^b^	USDA-NPGS
4	Ames 13,720	TUNDRI	United States, New Mexico^b^	USDA-NPGS
5	Ames 13,721	23 GR	United States, New Mexico^b^	USDA-NPGS
6	Ames 13,722	7ALC	United States, New Mexico^b^	USDA-NPGS
7	Ames 13,723	37TES	United States, New Mexico^b^	USDA-NPGS
8	Ames 13,724	18 GR	United States, New Mexico^b^	USDA-NPGS
9	Ames 13,725	46TES	United States, New Mexico^b^	USDA-NPGS
10	Ames 13,726	49ALC	United States, New Mexico^b^	USDA-NPGS
11	Ames 13,727	38TES	United States, New Mexico^b^	USDA-NPGS
12	Ames 13,728	27 GR	United States, New Mexico^b^	USDA-NPGS
13	Ames 13,729	10 GR	United States, New Mexico^b^	USDA-NPGS
14	Ames 13,730	1ESP	United States, New Mexico^b^	USDA-NPGS
15	Ames 13,731	42TES	United States, New Mexico^b^	USDA-NPGS
16	Ames 13,732	40TES	United States, New Mexico^b^	USDA-NPGS
17^a^	Ames 13,733	20TES	United States, New Mexico^b^	USDA-NPGS
18	Ames 13,734	47TES	United States, New Mexico^b^	USDA-NPGS
19	Ames 13,735	17 GR	United States, New Mexico^b^	USDA-NPGS
20	Ames 13,736	30TES	United States, New Mexico^b^	USDA-NPGS
21	Ames 13,737	2 WANT	United States, New Mexico^b^	USDA-NPGS
22	Ames 13,738	26TES	United States, New Mexico^b^	USDA-NPGS
23	Ames 13,739	29TES	United States, New Mexico^b^	USDA-NPGS
24	Ames 13,740	50ALC	United States, New Mexico^b^	USDA-NPGS
25	Ames 13,741	54ALC	United States, New Mexico^b^	USDA-NPGS
26	Ames 13,742	20 GR	United States, New Mexico^b^	USDA-NPGS
27	Ames 13,743	ISLUGA	Chile	USDA-NPGS
28	Ames 13,744	409	United States, New Mexico^b^	USDA-NPGS
29	Ames 13,745	KASLAEA	United States, New Mexico^b^	USDA-NPGS
30	Ames 13,746	PISON	United States, New Mexico^b^	USDA-NPGS
31	Ames 13,747	APELAWA	Bolivia	USDA-NPGS
32	Ames 13,748	COPACABANA	United States, New Mexico^b^	USDA-NPGS
33	Ames 13,749	32ALC	United States, New Mexico^b^	USDA-NPGS
34	Ames 13,750	31TES	United States, New Mexico^b^	USDA-NPGS
35	Ames 13,751	21 GR	United States, New Mexico^b^	USDA-NPGS
36	Ames 13,752	23TES	United States, New Mexico^b^	USDA-NPGS
37	Ames 13,753	16 GR	United States, New Mexico^b^	USDA-NPGS
38	Ames 13,754	52ALC	United States, New Mexico^b^	USDA-NPGS
39	Ames 13,755	43ALC	United States, New Mexico^b^	USDA-NPGS
40	Ames 13,756	3 UISE	United States, New Mexico^b^	USDA-NPGS
41	Ames 13,757	53ALC	United States, New Mexico^b^	USDA-NPGS
42	Ames 13,758	29TES	United States, New Mexico^b^	USDA-NPGS
43	Ames 13,759	20ALC	United States, New Mexico^b^	USDA-NPGS
44	Ames 13,760	75P	United States, New Mexico^b^	USDA-NPGS
45	Ames 13,761	47TES	United States, New Mexico^b^	USDA-NPGS
46	Ames 13,762	47TES	United States, New Mexico^b^	USDA-NPGS
47	NSL 86628	537 BK60-B	United States, Maryland	USDA-NPGS
48	NSL 86649	PLANT VIRUS	United States, South Carolina	USDA-NPGS
49	NSL 91567	PLANT VIRUS	United States, New York	USDA-NPGS
50	NSL 92331	JAPANESE STRAIN	United States, Washington	USDA-NPGS
51	PI 433232	-	Chile, Groben	USDA-NPGS
52	PI 470932	Pasan Ralle	Bolivia, La Paz	USDA-NPGS
53	PI 476820	Santa Elena 7	Mexico, Chapingo	USDA-NPGS
54	PI 478408	R-64	Bolivia, La Paz	USDA-NPGS
55	PI 478411	R-67	Bolivia, La Paz	USDA-NPGS
56	PI 478414	R-70	Bolivia, La Paz	USDA-NPGS
57	PI 478415	R-71	Bolivia, La Paz	USDA-NPGS
58	PI 478418	R-132	Bolivia, Potosi	USDA-NPGS
59	PI 510532	Quinoa de Quiaca	Peru, Puno	USDA-NPGS
60^a^	PI 510533	K’ello Quinoa (Quechua)	Peru, Puno	USDA-NPGS
61	PI 510534	Mezclada Tres Variedades (Span.)	Peru, Puno	USDA-NPGS
62	PI 510536	RB-35	Peru, Puno	USDA-NPGS
63	PI 510537	RB-52	Peru, Puno	USDA-NPGS
64^a^	PI 510538	Jaro Juira (Aymara), Quinoa Am	Peru, Puno	USDA-NPGS
65	PI 510539	RB-57	Peru, Puno	USDA-NPGS
66	PI 510540	Grande (Span.)	Peru, Puno	USDA-NPGS
67	PI 510541	Blanca de Grano Grande (Span.)	Peru, Puno	USDA-NPGS
68	PI 510542	Villa Juira (Aymara), Quinoa R	Peru, Puno	USDA-NPGS
69	PI 510544	Juira Sajama (Aymara), Quinoa	Peru, Puno	USDA-NPGS
70	PI 510545	Ccankolla (Aymara), Quinoa Saj	Peru, Puno	USDA-NPGS
71	PI 510547	Ara Juira (Aymara), Quinoa Sil	Peru, Puno	USDA-NPGS
72	PI 510548	Yulaj Q’anq’olla (Quechua), Qu	Peru, Puno	USDA-NPGS
73	PI 510549	Yulaj K’oyto (Quechua), Quinoa	Peru, Puno	USDA-NPGS
74	PI 510551	Quinua (Quechua), Quinoa var.	Peru, Puno	USDA-NPGS
75	PI 584524	QQ056	Chile, Chillan	USDA-NPGS
76	PI 596293	COLORADO 407D	United States, Colorado	USDA-NPGS
77	PI 596498	Rosa Junin	Peru, Cuzco	USDA-NPGS
78	PI 614002	Ames 10,334	Bolivia, Cochabamba	USDA-NPGS
79	PI 614880	QQ065	Chile,Los Lagos	USDA-NPGS
80^a^	PI 614881	QQ95	Argentina, Jujuy	USDA-NPGS
81	PI 614882	QQ67	Chile, La Araucania	USDA-NPGS
82	PI 614883	QQ101	Argentina, Jujuy	USDA-NPGS
83	PI 614885	QQ57	Chile, Bio-Bio	USDA-NPGS
84	PI 614886	QQ74	Chile, Maule	USDA-NPGS
85	PI 614887	QQ63	Chile, Bio-Bio	USDA-NPGS
86^a^	PI 614888	QQ61	Chile, Bio-Bio	USDA-NPGS
87	PI 614889	QQ59	Chile, Bio-Bio	USDA-NPGS
88	PI 614901	CQ101	Bolivia, Oruro	USDA-NPGS
89	PI 614902	CQ102	Bolivia, Oruro	USDA-NPGS
90	PI 614903	CQ103	Bolivia, Oruro	USDA-NPGS
91	PI 614904	CQ104	Bolivia, Oruro	USDA-NPGS
92	PI 614905	CQ105	Bolivia, Oruro	USDA-NPGS
93	PI 614906	CQ106	Bolivia, Oruro	USDA-NPGS
94	PI 614907	CQ107	Bolivia, Oruro	USDA-NPGS
95	PI 614909	CQ109	Bolivia, Oruro	USDA-NPGS
96	PI 614910	CQ110	Bolivia, Oruro	USDA-NPGS
97	PI 614911	CQ111	Bolivia, Oruro	USDA-NPGS
98	PI 614912	CQ112	Bolivia, Oruro	USDA-NPGS
99	PI 614913	CQ113	Bolivia, Oruro	USDA-NPGS
100	PI 614914	CQ114	Bolivia, Oruro	USDA-NPGS
101	PI 614915	CQ115	Bolivia, Oruro	USDA-NPGS
102	PI 614916	CQ116	Bolivia, Oruro	USDA-NPGS
103	PI 614917	CQ117	Bolivia, Oruro	USDA-NPGS
104	PI 614918	CQ118	Bolivia, Oruro	USDA-NPGS
105	PI 614919	CQ119	Bolivia, Oruro	USDA-NPGS
106	PI 614921	CQ121	Bolivia, La Paz	USDA-NPGS
107	PI 614922	Sayana	Bolivia, La Paz	USDA-NPGS
108	PI 614923	Jamiri	Bolivia, La Paz	USDA-NPGS
109^a^	PI 614924	CQ124	Bolivia, La Paz	USDA-NPGS
110	PI 614925	CQ125	Bolivia, La Paz	USDA-NPGS
111	PI 614927	CQ 127	Bolivia, La Paz	USDA-NPGS
112	PI 614928	CQ 128	Bolivia, La Paz	USDA-NPGS
113	PI 614929	CQ 129	Bolivia, La Paz	USDA-NPGS
114	PI 614930	CQ 130	Bolivia, La Paz	USDA-NPGS
115	PI 614931	CQ 131	Bolivia, Oruro	USDA-NPGS
116^a^	PI 614932	CQ 132	Bolivia, Oruro	USDA-NPGS
117	PI 614933	CQ 133	Bolivia, Oruro	USDA-NPGS
118	PI 614935	CQ 135	Bolivia, Oruro	USDA-NPGS
119	PI 614936	CQ136	Bolivia, Oruro	USDA-NPGS
120	PI 614938	CQ139	Bolivia, Oruro	USDA-NPGS
121	PI 634917	Pichilemu	Chile, Bio-Bio	USDA-NPGS
122^a^	PI 634918	Baer	Chile, Bio-Bio	USDA-NPGS
123	PI 634919	Pichaman	Chile, Bio-Bio	USDA-NPGS
124^a^	-	Riobamba	The Netherlands^c^	Private
125^a^	-	Atlas	The Netherlands^c^	Private
126	-	Pasto	The Netherlands^c^	Private
127	-	-	China^c^	Private
128	-	-	China^c^	Private
129	-	-	Germany^c^	Private

^a^Used for genomic re-sequencing

^b^Quinoa accessions donated by Emigdio Ballón were assigned to “United States, New Mexico”

^c^Collection place

### DNA sample preparation and re-sequencing

Based on morphological features and variations, 11 quinoa accessions were selected and grown at the Luhe Experimental Station of Jiangsu Academy of Agricultural Science (JAAS) for de novo genomic re-sequencing (Table 1). Whole young plants above the ground were collected and quickly frozen in liquid nitrogen. Total DNA from 10 individuals for each line was extracted using the plant DNA extraction kit from Qiagen. The sequencing libraries (2 × 250 bp for Riobamba and 2 × 150 bp for the other ten accessions) were constructed following the manufacturer’s instructions (Illumina Inc.). Paired-end sequencing was conducted on an Illumina HiSeq 2500 sequencer at BerryGenomics Company.

### InDel and SNP calling

The 2 × 250 bp cleaned reads were assembled with SOAPdenovo to generate longer sequences for Riobamba de novo scaffold assembly [39]. First, a De Bruijn graph was constructed using an optimal 61 kmer size. Non-repetitive contigs within a graph were subsequently assembled into scaffolds based on mapping information from single-end reads. Scaffolds shorter than 100 bp and erroneous connections were filtered out. The assembled scaffold sequences from the accession Riobamba were used as a reference for SNP and InDel calling. Paired-end sequencing reads from the other ten accessions were mapped to the Riobamba reference scaffold sequence with BWA using default parameters [40]. The unmapped and non-unique reads were filtered out using SAMtools with MAQ ≥ 30 [41]. The InDelRealignment method was employed to avoid InDel false positives. SNP and InDel detection were performed by employing GATK with HaplotypeCaller mode [42]. To reduce the false positive rate, filters were applied such that bi-allelic loci with depth greater than 10 reads and confidence score greater than 30 remained.

### Dimorphic InDel marker screening

Dimorphic InDel marker discovery was conducted using the high-throughput and genome-wide InDel marker development software mInDel [43]. mInDel identifies long InDel polymorphisms and develops genetic markers independent of a reference genome. According to the mInDel procedure, de novo-assembled sequences from ten quinoa accessions were mapped to the reference Riobamba assembly for InDel calling. After primer design, the optimal dimorphic InDel markers predicted by mInDel were validated by agarose gel electrophoresis (AGE) and polyacrylamide electrophoresis (PAGE). The physical positions of the validated primers in the quinoa draft genome were obtained using BLASTN against the scaffold sequences in Cqu_r1.0.

### Genotyping the quinoa population

In total, 129 quinoa lines were planted at the Luhe Experimental Station of JAAS. Methods for sample collection and DNA extraction followed that from genomic re-sequencing. In total, 147 markers were used for genotyping, which included 85 self-developed and validated dimorphic InDels, 14 screened genomic SSRs (gSSRs) [25] and 48 SSRs derived from expressed sequence tag (EST) libraries (EST-SSRs) [23] that all gave reproducibly amplified products and could be confidently scored. Thirty-eight dimorphic InDel markers were detected as large variations by 3% AGE, and the remaining 109 markers were detected by 12% PAGE. Each PCR reaction contained a 25 μl total volume consisting of 2 μl template DNA, 2.5 μl 10× PCR buffer (Mg^2+^ free), 2.5 μl 25 mM MgCl_2_, 2.5 μl 10 mM dNTPs, 2 μl 100 μM primers, 0.2 μl 5 U/μl Tag and 13.3 μl ddH_2_O. The following PCR conditions were used for amplification: (1) a pre-denaturation initial step at 95 °C for 3 min; (2) 38 cycles of 95 °C for 40 s, 58 °C for 40 s, 72 °C for 40 s; and (3) 72 °C for 5 min.

### Genetic diversity analysis

The POWERMARKER 3.25 software was used to provide basic summary statistics [44]. Basic summary statistics included the total number of alleles, major allele frequency, genetic diversity, heterozygosity, inbreeding coefficient, and the polymorphism information content (PIC). According to the PIC value, markers were classified as highly informative (PIC >0.5), moderately informative (0.25 < PIC <0.5), and slightly informative (PIC <0.25) [45]. The Euclidean distance between two accessions was calculated with the POWERMARKER 3.25. A transformed squared Euclidean distance matrix was used as input for the Arlequin 3.5 software for the analysis of molecular variance (AMOVA) [46]. The statistical significance of each variance component and population pairwise fixation index (F_ST_) were assessed based upon 20,022 data permutations.

### Population structure analysis

Genetic structure analysis of the accessions was performed with the STRUCTURE 2.3.4 software [47, 48] and by Principle Component Analysis (PCA) with the TASSEL 2.1 software (http://www.maizegenetics.net/). The STRUCTURE software employs a Bayesian, model-based clustering algorithm to assign individuals to groups with a predetermined number (*K*) in a manner that minimizes Hardy-Weinberg and linkage disequilibrium within each group. Using the admixture model with no prior populations indicated, ten independent runs for each *K* ranging from 1 to 10 were performed and 10,000 iterations were employed for estimation after a 10,000 iteration burn-in period. An ad hoc statistical △*K* based on the rate of change in the log probability between successive *K* values was calculated to estimate the subgroups and the best *K* [49]. The subgroups differentiated by PCA were also considered comprehensively for the terminal subgroup conformation.

### Phylogenetic analysis

Nei’s genetic distance (1983) matrix of 129 quinoa accessions based on 147 markers was calculated by the POWERMARKER software package 3.25 [50]. Pairwise genetic distances for the 10 re-sequenced accessions excluding the reference Riobamba were calculated based on the filtered genomic SNPs and InDels via identity by state (IBS) similarity in the TASSEL 5.0 program (http://www.maizegenetics.net/). Phylogenetic trees were constructed using the neighbor joining (NJ) algorithm in the MEGA 7.0.14 software [51]. Pearson’s product-moment correlation coefficient (*r*) was used to investigate the relationships between the pairwise genetic distance matrix for the 10 re-sequenced accessions.

### Identification of a core set of quinoa germplasms

The line selection algorithm was used to identify the optimal core set of 129 quinoa accessions based upon simulated annealing [52]. Based on genotypic data, the analysis was performed by the POWERMARKER software package 3.25. The core set sample size was set to range from 3 to 120, with one run per sample size. To increase the probability of finding the global maximum, the following parameters were used: (1) swapping number *R* = 3000, (2) cooling coefficient *ρ* = 0.95, and (3) initial annealing temperature *T*_*0*_ = 1.

## Results

### SNP and InDel variation

**Fig. 1:**
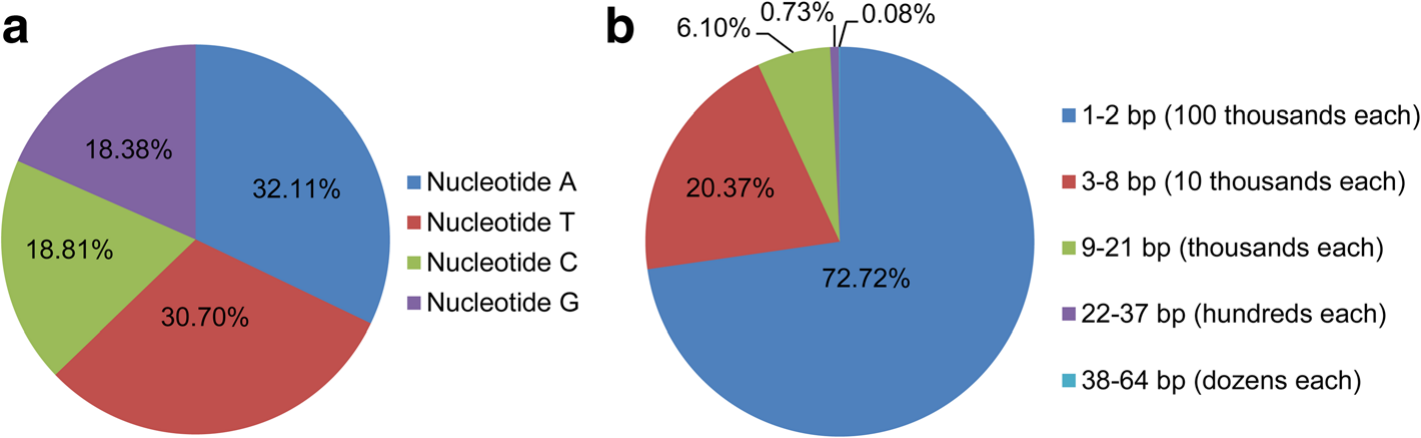
Nucleotide content and filtered InDel length distributions. **a** Nucleotide content and (**b**) filtered InDel distributions

To identify variations in the quinoa genome, 11 quinoa accessions representing geographical adaption within species were selected for Illumina de novo paired-end sequencing. In total, 0.44 billion paired-end reads were generated; these reads had a coverage depth of approximately 7× to 8× the quinoa genome (approximately 23× for Riobamba) based on the previously estimated genome size of 1448 Mb (Additional file 1: Table S1). De novo assembly of the Riobamba accession resulted in 4,890,868 contigs and 4,147,776 scaffolds, with the longest sequence being 36,019 bp and 65,344 bp and having N50 sizes of 757 bp and 2667 bp, respectively (Additional file 2: Table S2). The assembled scaffolds were 1453 Mb in size. Nucleotide statistics on the assembled scaffolds showed that the GC content (37.19%) was obviously lower than the AT content (62.81%) (Fig. 1a; Additional file 2: Table S2). In total, 8,441,022 filtered bi-allelic SNPs and 842,783 filtered InDels were generated between each of the ten accessions and Riobamba using the assembly based method. Based on the assembled scaffold size, the SNP and InDel densities in the quinoa genome were estimated to be 5.81 and 0.58 per kilobase (kb) each (Additional file 2: Table S2). The majority of InDels was small and ranged from 1 to 2 bp (72.72%) and 3–8 bp (20.37%), whereas InDels longer than 8 bp had the smallest proportion (6.91%) (Fig. 1b; Additional file 3: Table S3). Statistical analysis of the InDels revealed that InDel length showed a highly significant and negative correlation with the InDel number (*r* = −0.335, *P* = 0.008).

### Dimorphic InDel marker analysis

A total of 90 InDel markers were selected randomly from the best-scored dimorphic marker set predicted by the mInDel software. After AGE and PAGE validation, four markers that could not amplify the major allele band, and one marker shown to be monomorphic was abandoned. The remaining 85 InDel markers were confidently scored and exhibited dimorphism (Additional file 4: Table S4 and Additional file 5: Fig. S1). Based on the BLAST results against the quinoa draft genome, 71 of the InDel markers were placed, of which 67 showed complete consistencies between their predicted length and the BLAST length on the draft genome (Additional file 4: Table S4 and Additional file 5: Fig. S1). Among the four markers that showed length differences with the draft genome, three had only 1–8 nucleotide differences, and one had a large difference with a magnitude of thousands of nucleotides (Additional file 4: Table S4 and Additional file 5: Fig. S1). Together with the 14 gSSRs and 48 EST-SSRs, a total of 147 markers were used for genotyping across the 129 quinoa accessions (Additional file 6: Table S5). Of the 362 alleles detected, one allele was found to be uniquely present in only one accession, and 41 alleles were found to be rare (present in <5% accessions) (Table 2). Due to the dimorphic nature of InDel markers, the unique and rare alleles were expected to be difficult to detect in this sample population. PIC statistics revealed that most of the dimorphic InDel markers were moderately informative and that the average PIC of the dimorphic InDel markers was equivalent to that of EST-SSRs and slightly lower than that of gSSRs (Table 2). Considering all three marker types, the correlation analysis showed that the allele number was significantly correlated with PIC (*r* = 0.668, *P* < 0.001).

**Table 2:** Summary of the alleles and PIC values of the InDel, gSSR and EST-SSR markers

Marker type	Markernumber		Alleles		Informative type			PIC	*r* ^a^
		Total	Unique	Rare	Slight	Moderate	High		
InDel	85	170	0	0	1	84	0	0.36	-
gSSR	14	46	0	5	3	6	5	0.41	*0.840* ^***^
EST-SSR	48	146	1	36	13	23	12	0.36	*0.722* ^***^

^a^Correlation between the allele number and PIC value for each marker

^***^ Significant difference at *P* < 0.001 level

### Population structure and genetic diversity

**Fig. 2:**
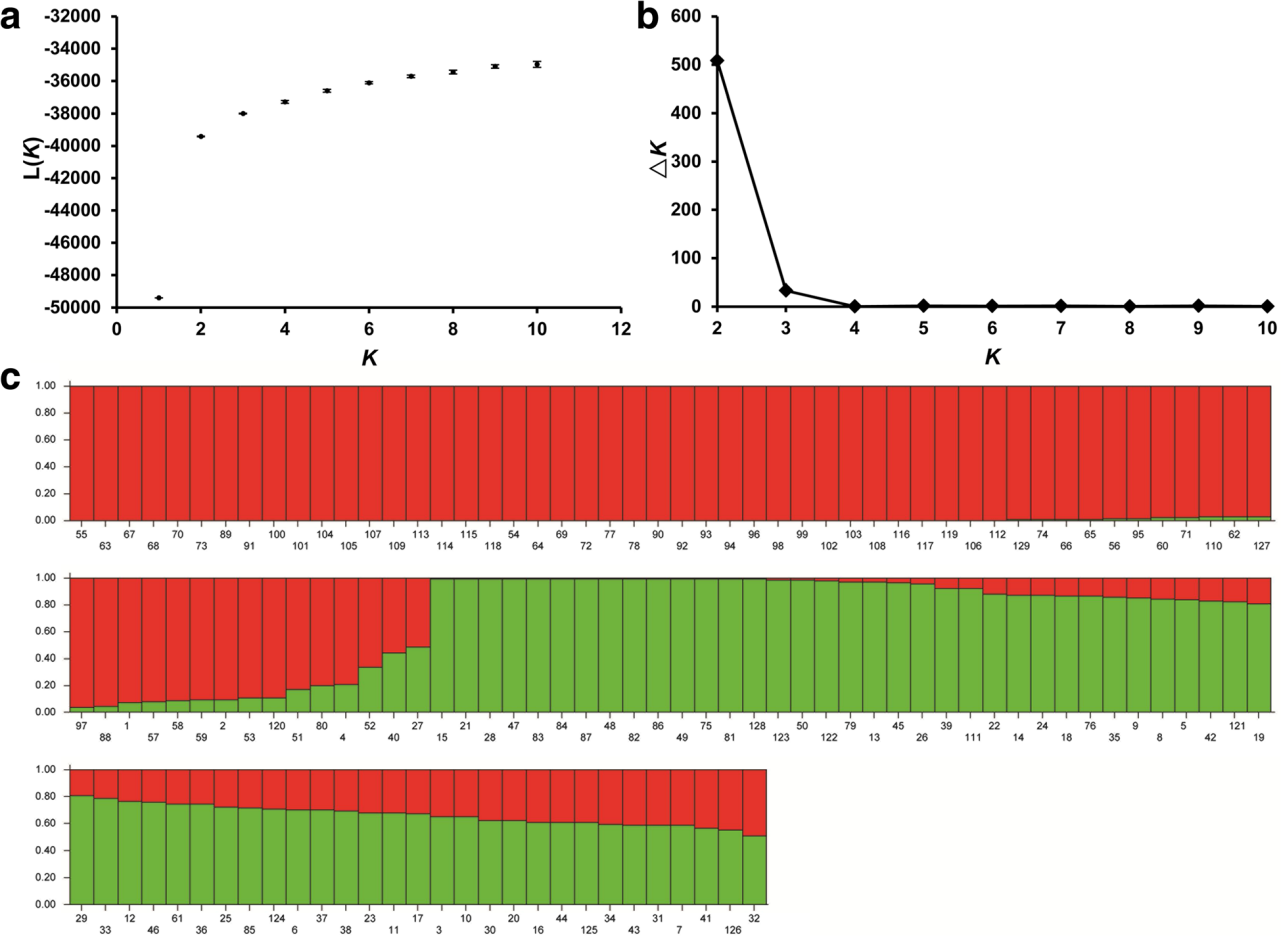
Population structure of the 129 quinoa accessions analyzed by STRUCTURE. **a** L(*K*) (log probability of data, mean ± SD) over ten runs for *K* ranging from 1 to 10. **b** Estimation of the optimal group number using △*K*. **c** *Q*-plot of the population structure. Each quinoa accession is represented by a *vertical bar*. The numbers on the *y*-axis indicate the membership coefficient (*Q*), while the numbers on the *x*-axis indicate the serial number of each accession

**Fig. 3:**
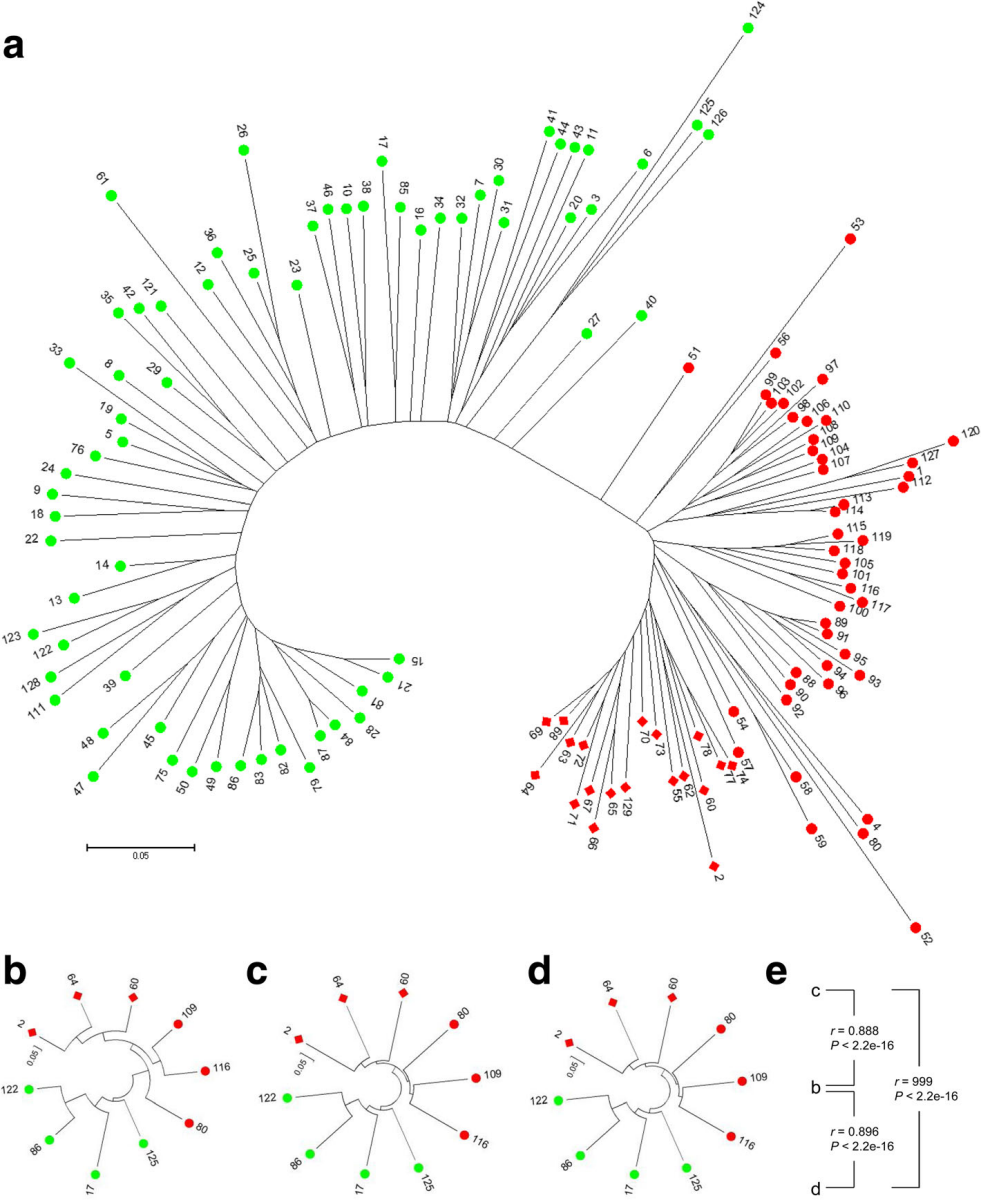
Quinoa phylogenetic trees using the NJ algorithm. The quinoa accessions are color-coded based on the groups identified by STRUCTURE and represented by their serial number. **a** The NJ tree with the 129 quinoa accessions based on Nei’s genetic distance (1983) calculated from the 147 markers. **b-d** NJ trees for the 10 re-sequenced quinoa accessions based on (**b**) Nei’s genetic distance (1983) calculated from the 147 markers, (**c**) IBS similarity calculated from the genomic filtered SNPs, and (**d**) InDels. **e** Correlations between the three NJ trees for the 10 re-sequenced quinoa accessions

**Fig. 4:**
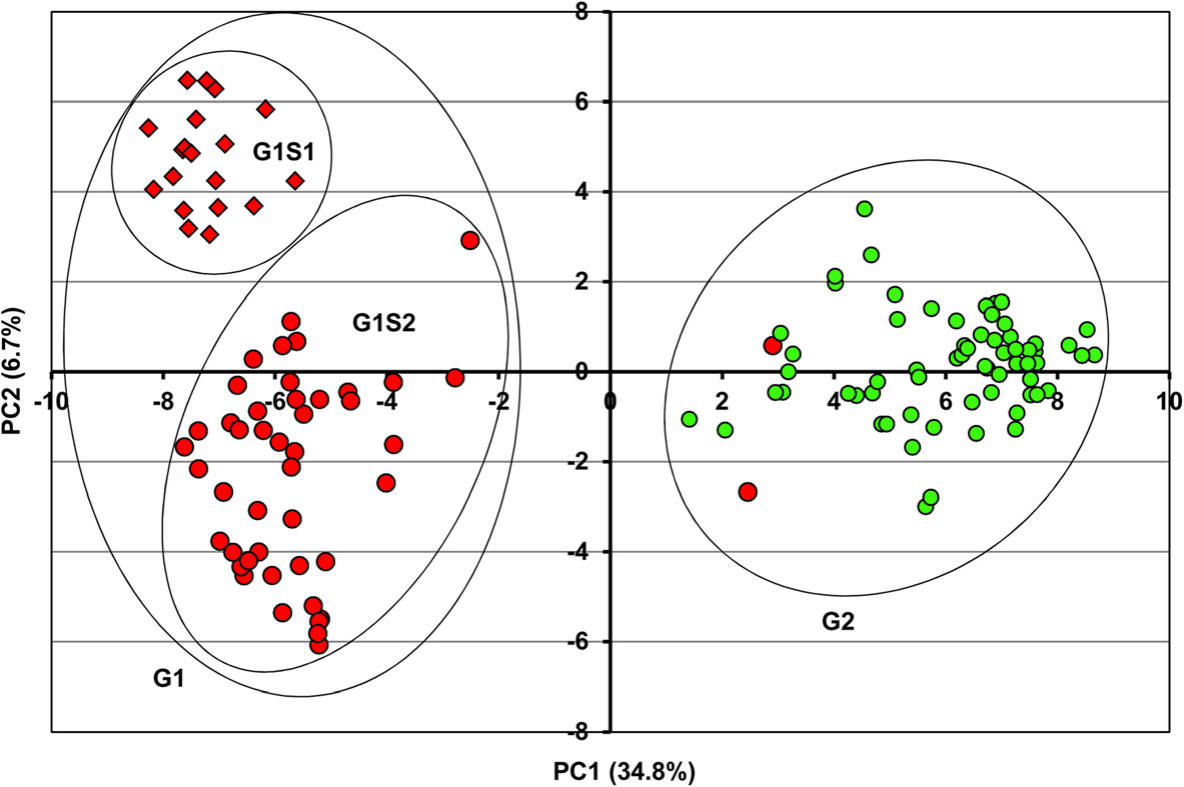
Scatter plot from the PCA for the 129 quinoa accessions. The color and shape scheme is the same as that for the NJ analysis

Population structure analysis was performed on the complete 129 quinoa accessions using STRUCTURE based on the 147 markers. Both L(*K*) and △*K* values demonstrated that the two groups were the optimal classification for these quinoa accessions (Figs. 2a and b). The *Q*-plot output from STRUCTURE presented our grouping results (Fig. 2c). NJ analysis for this quinoa set generated two major branches, showing consistency with the STRUCTURE results (Fig. 3a). Only accessions 27 and 40 were clustered into different groups between these two methods. To validate the phylogenetic results, NJ trees of the ten re-sequencing accessions based on the millions of filtered genomic SNPs and InDels (minor allele frequency ≤ 0.05 and missing rate ≥ 0.1) were compared with NJ tree based on the 147 markers (Fig. 3b-e). The pairwise comparisons of the genetic distances exhibited strong statistical correlations between the three NJ trees, demonstrating that the grouping results from the phylogenetic analysis based on the 147 markers is highly reliable. Additionally, the grouping results by PCA were consistent with the NJ analysis (Fig. 4). The first two PCA axes accounted for 41.5% of the total variation observed in the 129 quinoa samples. PC1 explained 34.8% of the overall variation and separated the whole accessions into two major groups named G1 (red) and G2 (green and two reds corresponding to 27 and 40) (Fig. 4). Within G1, two subgroups named G1S1 and G1S2 were clearly displayed based on PC2, which explained 6.7% of the total variation (Fig. 4). These groups were the best for the 129 quinoa samples when combining the STRUCTURE, phylogenetic tree and PCA results.

Our grouping results agree with previous reports that there are two main quinoa groups in South America, the Andean highland type (G1) and the Chilean coastal type (G2), where the Andean highland type is further classified into northern highland (G1S1) and southern highland (G1S2) subgroups. The level of genetic diversity of G2 (0.38), which includes most accessions from Chile and “United States, New Mexico”, was higher than G1 (0.33) with most accessions from Peru and Bolivia (Table 3). Within G1, the genetic diversity of subgroup G1S2 (0.32), with most accessions from Bolivia, was higher than G1S1 (0.27), with most accessions from Peru (Table 3). Both subgroups were expected to have lower levels of genetic diversity than the main G1 and G2 groups. This finding was also supported by the average genetic distance between the groups and subgroups (Table 4).

**Table 3:** Summary statistics for the quinoa groups

Statistics	Overall	G1	G2	G1S1	G1S2
Sample size	129	63	66	19	44
Total number of alleles	362	353	336	304	345
Number of alleles per locus	2.46	2.40	2.29	2.07	2.35
Genetic diversity	0.45	0.33	0.38	0.27	0.32
Heterozygosity	0.04	0.04	0.04	0.04	0.04
PIC	0.36	0.28	0.31	0.23	0.27
Inbreeding coefficient	0.92	0.89	0.90	0.86	0.89

**Table 4:** Genetic distance estimates between the groups and subgroups

Group	G1	G2	G1S1	G1S2
G1		*0.705* ^***^		
G2	0.411		*0.752* ^***^	*0.695* ^***^
G1S1		0.435		*0.353* ^***^
G1S2		0.402	0.220	

Top diagonal with bold font is pairwise F_ST_ among the groups and subgroups, and the bottom diagonal is the average of Nei’s genetic distance (1983)

^***^ Significant difference at *P* < 0.001 level

From the STRUCTURE analysis, the G1 and G2 groups showed a certain degree of gene exchange (Fig. 2c). Most accessions from Peru and Bolivia in G1 are unmixed, with only a small number of accessions gaining genome content from G2. In G2, Chilean coastal accessions are unmixed and the majority of accessions marked “United States, New Mexico” included genome contents from G1. To further examine the gene flow, the population structure of the three groups (G1S1, G1S2, and G2) was evaluated (Additional file 7: Fig. S2 and Additional file 8: Fig. S3). The results revealed that gene flow between the subgroups was more frequent than between the two G1 subgroups and G2, whereas the gene content of the two subgroups both contributed to the genomes of admixed individuals in G2.

AMOVA was conducted to investigate genetic relationships among quinoa groups. The results showed that approximately 70% of the total variation was due to among-group differences, and the remaining 30% of variation was due to diversity within the groups or subgroups (Table 5). The pairwise population differentiation estimate showed a highly significant F_ST_ value (0.705) between groups G1 and G2, suggesting large genetic differentiation between the Andean highland and Chilean coastal quinoa types (Table 4). As expected, similar highly significant F_ST_ values were observed between G2 and each of the G1S1 and G1S2 subgroups (Table 4). In contrast, a relatively lowly significant F_ST_ value (0.353) was found between the two subgroups, suggesting a low level of differentiation between the accessions in the northern and southern highland subtypes.

**Table 5:** AMOVA for the quinoa accessions between and within groups (subgroups)

Source of variation	*df*	Sum of squares	Variance components	Percentage of variation (%)
Between populations ^a^	1	9.335	*0.144* ^***^	70.51
Within populations	127	7.644	0.061	29.49
Total	128	16.979	0.204	
Among populations ^b^	2	10.01	*0.128* ^***^	69.80
Within populations	126	6.969	0.055	30.20
Total	128	16.979	0.183	

^a^Between the group G1 and G2

^b^Among the subgroup G1S1, G1S2, and group G2

^***^ Significant difference at *P* < 0.001 level

### Core set of quinoa

**Fig. 5:**
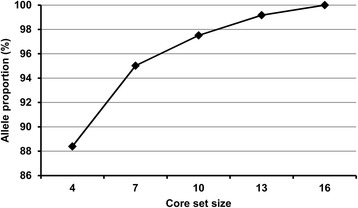
The maximum allele percentage captured for a core set with a given sample size

The POWERMARKER software was used to identify a core set of quinoa based on the genotyping data with 85 InDel, 14 gSSR and 48 EST-SSR markers. Selection was made between the Andean highland type G1 and Chilean coastal type G2, with at least one accession selected for each group. The smallest sample set consisted of only four accessions and accounted for 88% of total alleles, suggesting the high genetic diversity of these accessions (Fig. 5). When the sample size increased to seven, 95% of alleles was captured (Fig. 5). To obtain a sample representing 100% of the 362 alleles, a sample size of 16 accessions was required (Fig. 5). Overall, for these 129 quinoa accessions, a small number of individuals retained the most frequent alleles as well as the entire allelic diversity. The list of complete accessions in sets with different sizes is shown in Table 6.

**Table 6:** Core sets of the 129 quinoa accessions identified using POWERMARKER

Core set size		4	7	10	13	16
Accessions (group)	PI 478411 (G1S1)		Y			
	PI 510538 (G1S1)		Y	Y	Y	Y
	PI 510539 (G1S1)	Y	Y	Y	Y	Y
	PI 510541 (G1S1)					Y
	PI 510542 (G1S1)					Y
	PI 510544 (G1S1)				Y	Y
	PI 510548 (G1S1)				Y	Y
	PI 614881 (G1S2)	Y		Y		
	PI 614902 (G1S2)					Y
	PI 614904 (G1S2)				Y	
	PI 614905 (G1S2)				Y	
	PI 614906 (G1S2)		Y			
	PI 614909 (G1S2)				Y	Y
	PI 614911 (G1S2)			Y		Y
	PI 614912 (G1S2)					Y
	PI 614913 (G1S2)					Y
	PI 614914 (G1S2)		Y			
	PI 614928 (G1S2)		Y	Y	Y	Y
	PI 614929 (G1S2)			Y	Y	Y
	PI 614932 (G1S2)			Y		
	PI 614933 (G1S2)					Y
	Ames 13,725 (G2)	Y	Y	Y	Y	
	Ames 13,740 (G2)				Y	
	Ames 13,747 (G2)			Y	Y	Y
	Ames 13,754 (G2)					Y
	NSL 92331 (G2)	Y				
	PI 584524 (G2)			Y		
	PI 634918 (G2)				Y	
Allele number		320	350	356	361	362

Y represents that accessions selected in corresponding core set

## Discussion

### Genomic variation of quinoa

To better understand the genetic variation in quinoa, de novo re-sequencing was employed to analyze 11 morphologically distinct quinoa accessions representing 129 germplasm lines mainly from the USDA-NPGS. Grouping analysis demonstrated that these 11 quinoa accessions extensively represented the northern highland (three accessions), southern highland (three accessions) and Chilean coastal types (five accessions) in the main planting area of South America. Based on the scaffolds from the Riobamba accession, the assembled quinoa genome has a size of 1453 Mb, which agrees well with previously reported estimates on quinoa genome size, such as 1448 Mb by cytometry analysis [17] and 1.5 gigabases (Gb) from Cqu_r1.0 [15] and 1.39 Gb from *Chenopodium quinoa v1.0* [21] calculated using assembled scaffolds. In total, 8,441,022 filtered bi-allelic SNPs and 842,783 filtered InDels were identified. The density of SNP and InDel polymorphisms distributed in the quinoa genome was estimated to be 5.81 and 0.58 per kb, respectively, which is much less than other crop species such as maize, where SNPs and InDels occur every 79 and 309 bp, respectively [53]. In a previous study, 14,178 putative SNPs were discovered using a genomic reduction protocol against eight quinoa accessions representing a broad geographical distribution with average SNP distance of 2160 bp [24]. The large discrepancy in SNP density appears to be caused by different SNP calling strategies. Compared with the genomic reduction protocol, de novo genomic re-sequencing is more direct and reliable for genomic variation investigations. Because the genomic reduction process could reduce the DNA complexity of quinoa nearly 52-fold, the selective small size is insufficient to represent the whole genome and may lead to underestimation of the genomic variation. Additionally, our results revealed a 37.19% GC content in the quinoa genome, which is congruent with the 36.9% GC content in the Cqu_r1.0 quinoa draft genome [15] and slightly lower than the 43% GC content identified using a relatively smaller size sample of 100 ESTs and 35 quinoa genomic sequences [14]. In terms of InDel polymorphisms, the most prevalent types in the quinoa genome are small InDels ranging from 1 to 2 bp (72.72%) to 3–8 bp (20.37%). The number of 1–6 bp InDels, the most-studied InDel type, is 750,731, making up 89.08% of all the InDels in the quinoa genome, which is a little less than in soybean and twice that of maize [54, 55]. It should be noted that trinucleotide InDels could result in a shift in open reading frames and cause functional changes in their corresponding genes. For example, a trinucleotide InDel was found in *Spiral2*, a key gene related to directional cell elongation in Arabidopsis [56]. Although many InDels (58,186) are trinucleotide InDels in quinoa, frameshift may not cause damage due to the allotetraploid nature of quinoa; as a frameshift in a gene in one subgenome may be compensated for by an allele from another subgenome. In addition to SNPs and InDels, copy number variations (CNVs) and presence-absence variations (PAVs), which are often associated with agronomic traits, are frequently analyzed in genomic variation [54, 57–59]. However, the precise characterization of these variations is dependent on a complete genome of a species.

### SSR and InDel markers

Molecular markers are important tools for marker-assisted selection (MAS), germplasm conservation and core germplasm selection for modern breeding. Among the types of genetic markers, SSR is a widely used marker type. However, in quinoa, only 430 polymorphic gSSR markers and 49 polymorphic EST-SSR markers were identified [23, 25, 27]. According to previous studies, some highly polymorphic genomic SSR markers with several potential alleles per locus were extensively used for genetic analysis in quinoa [31–33]. These highly polymorphic SSRs could result in non-specific amplifications and cause confusion in genotyping scoring, especially for allotetraploid species such as quinoa. Therefore, new markers should be developed to better serve quinoa researchers. In this study, we validated 85 of 90 newly developed dimorphic InDel markers selected from the best predictions based on the de novo genomic assembled sequences. High congruency between the lengths of these InDel markers with the Cqu_r1.0 quinoa draft genome supported the reliability of our analysis. However, 14 InDel markers were not anchored in Cqu_r1.0, and one anchored InDel marker was found to have a large length discrepancy, which could be caused by potential PAV in the draft genome accession Kd or incompletely assembled scaffolds in the draft genome. Because InDels could affect gene functions by causing the gain or loss of a stop codon and/or frameshift, InDels can be developed into functional markers that would be particularly useful for MAS.

### Genetic diversity and differentiation

The population structure and diversity of quinoa at the phenotypical or molecular level have been reported in several previous studies [29–31, 33, 60, 61]. By combining the STRUCTURE, phylogenetic tree and PCA results, we found that a grouping of two distinct major types, Andean highland and Chilean coastal groups, and two subgroups within the Andean highland group, northern and southern highland subgroups, is obvious based on the 129 quinoa samples. The strong genetic differentiations between the groups and subgroups were confirmed by the high F_ST_ values. These grouping results are congruent with those from the previous two reports on partial quinoa accessions from the USDA using SSRs and SNPs [24, 31]. With the unique accession numbers assigned by the USDA, 86 accessions were found to be in the UPGMA phylogenic tree and Jaccard’s similarity coefficient based on 36 SSRs [31]. Of these, 14 quinoa germplasms displayed between-group and between-subgroup grouping difference. The small discrepancy may be attributed to the difference in marker types, marker numbers and grouping methods. Specifically, accession PI 476820, suggested as being *C. berlandieri* Moq. ssp. *Nuttalliae* [31], could not be excluded from *C. quinoa* according to our analysis. For accessions without origin information, grouping analysis is a feasible way to evaluate their identity. One Chinese quinoa germplasm and three Holland’s quinoa lines, including Riobamba, were suggested as being from Chilean coastal group. The other Chinese quinoa and one Germany germplasm may be linked to La Paz, Bolivia and Puno, Peru respectively. Additionally, accession PI 614886 (serial number 84), which was used for constructing the assembled genome of *Chenopodium quinoa v1.0*, was grouped into the Chilean coastal type, which is consistent with a previous report [21].

Our findings suggest a decrease in genetic diversity from the Chilean coastal to the northern area of the Andean highland. It appears that the quinoa germplasms from the Chilean coast have the highest level of genetic diversity in the Andean region, which is consistent with a previous report that alternatively attributed the high diversity to the outcross between lowland quinoa and the *C. album* and/or *C. hircinum* weed population [32]. However, this disagreed with the observation that the genetic diversity of the coastal lowland and northern highland regions was lower than the southern highland region [31], which supports the views that the southern highland regions near Lake Titicaca between Peru and Bolivia are the genetic diversity center for quinoa [30]. From the gene flow perspective, gene transfer between the northern and southern highland subgroups is higher than between the subgroups and the coastal group. This could explain the relatively low F_ST_ between the subgroups. Moreover, the quinoa germplasms donated to the USDA by Bolivian agronomist Emigdio Ballón have varying proportions of their genomes derived from the two subgroups, suggesting that these accessions may originate from the zones in between the southern highlands and Bio-Bio, Chile [31]. However, the co-occurrence of admixed and unadmixed quinoas in the Andean region of South America implies complex gene flow among different regions under the influence of natural and artificial selection.

### Genetic core germplasm for quinoa

The main objective of selecting a genetic core collection is to provide a smaller set of accessions that best represent the genetic variability of a broad germplasm. In quinoa, two studies have been reported on selecting a core set using morphological data on the Peruvian germplasm [37, 38]. In our study, by employing 147 molecular markers including InDels, gSSRs and EST-SSRs, core sets with different sample sizes representing different levels of allelic richness were established based on 129 quinoa lines representing the major cultivation regions in South America. A set of four accessions distributed in the northern and southern highland subgroups and Chilean coastal group can capture 88% of the alleles, while a core set of 16 accessions contributing to 37.5%, 50%, and 12.5% of the germplasm from the northern and southern highland subgroups and Chilean coastal group, respectively, is sufficient to capture all 362 alleles. These core sets of quinoa provide invaluable information for germplasm conservation and could be used to develop genetic populations to scan target loci and genes and for selecting parental accessions to improve breeding levels.

## Conclusions

We re-sequenced 11 quinoa germplasms representative of morphological diversity to gain knowledge on genomic variation in quinoa. Comparison of the assembled sequence revealed a large number of genomic variations, including SNPs and InDels with a frequency of 5.81 and 0.58 per kb, respectively. These variations demonstrated that the quinoa genome is highly variable. Based on the assembled data, dimorphic InDel markers of quinoa were predicted and validated. These novel InDel markers can be used for accurately genotyping allotetraploid quinoa to avoid the instability of genotyping scores. Using these markers, two main quinoa groups, the Andean highland type and the Chilean coastal type, were identified. The Andean highland type was further classified into the northern highland and southern highland subgroups. Strong genetic differentiations supported by high F_ST_ values were found between the groups and subgroups. A gradually decreasing tendency in genetic diversity from the Chilean coastal to the northern Andean highland was observed. Gene exchange between the subgroups was shown to be more frequent than between the two main groups. Furthermore, the selection of core set comprising varying quinoa accessions will be very useful for improving breeding levels and genetic research on quinoa.

## Additional files

Additional file 1: **Table S1.** Summary of the de novo re-sequencing results for the 11 quinoa germplasms. (XLSX 9 kb)

Additional file 2: **Table S2.** De novo-assembled data for the Riobamba accession. (XLSX 10 kb) (XLSX 9 kb)

Additional file 3: **Table S3.** Statistics on the filtered InDel lengths and number. (XLSX 9 kb)

Additional file 4: **Table S4.** The primer sequence, predicted PCR product length, and physical position of the validated dimorphic InDel markers. The bold font in the “BLAST length” column represents that the corresponding InDel marker displays a difference between its predicted length and the BLAST length. (XLSX 20 kb)

Additional file 5: **Fig. S1.** Validation of predicted dimorphic InDel marker. Each lane represents one quinoa genotype. Homozygous genotype was indicated as one amplification band, while heterozygous genotype was indicated as two amplification bands per lane. **a** Validation of a marker with a large PCR length difference by 3% AGE without DNA marker (48 lanes). Markers JAAS14 and JAAS67 were 56 bp and 100 bp, respectively, as predicted from PCR length differences. **b** Validation of a marker with a small PCR length difference by 12% PAGE with DNA marker (25 lanes). The predicted PCR lengths of marker JAAS78 are 200 bp and 166 bp, while that of JAAS85 are 231 bp and 209 bp. M represents DNA marker I with six DNA fragment sizes (600 bp, 500 bp, 400 bp, 300 bp, 200 bp, and 100 bp). (PDF 687 kb)

Additional file 6: **Table S5.** Genotyping of 129 quinoa samples using 85 dimorphic InDel markers. Quinoa germplasms are represented by their serial numbers in Table 1. Numbers 1 and 2 represent homozygous genotypes, 3 represents a heterozygous genotype, and 9 represents missing in the genotyping section. (XLSX 47 kb)

Additional file 7: **Fig. S2.** Population structure of the 129 quinoa accessions shown via STRUCTURE (*K* = 3). Each quinoa accession is represented by a *vertical bar*. Numbers on the *y*-axis indicate the membership coefficient, while the numbers on the *x*-axis indicate the serial number of each accession. The three groups are marked separately by red, green and blue colors. (PDF 104 kb)

Additional file 8: **Fig. S3.** The NJ tree of the 129 quinoa accessions based on Nei’s genetic distance (1983) calculated from the 147 markers. The quinoa accessions are color-coded based on the groups identified by STRUCTURE (*K* = 3). (PDF 163 kb)

## Abbreviations

AFLP: amplified fragment length polymorphism
AGE: agarose gel electrophoresis
AMOVA: analysis of molecular variance
BWA: Burrows-Wheeler Aligner
CNVs: copy number variations
EST: expressed sequence tag
EST-SSRs: SSRs derived from EST
GATK: Genome Analysis Toolkit
Gb: gigabase
gSSRs: genomic SSRs
IBS: identity by state
InDels: Insertions/Deletions
JAAS: Jiangsu Academy of Agricultural Science
kb: kilobase
MAS: marker-assisted selection
Mb: megabase
NJ: neighbor jointing
PAGE: polyacrylamide electrophoresis
PAVs: presence-absence variations
PCA: Principle Component Analysis
PIC: polymorphism information content
RAPD: random amplified polymorphic DNA
SNPs: single nucleotide polymorphisms
SSR: simple sequence repeat
USDA: the United States Department of Agriculture
USDA-NPGS: the United States Department of Agriculture-National Plant Germplasm System
