# Supplementary figures and images for: Development of novel InDel markers and genetic diversity in Chenopodium quinoa through whole-genome re-sequencing
Source: BMC Genomics. 2017 Sep 5;18:685. doi: 10.1186/s12864-017-4093-8 (PMC5584319; doi:10.1186/s12864-017-4093-8)

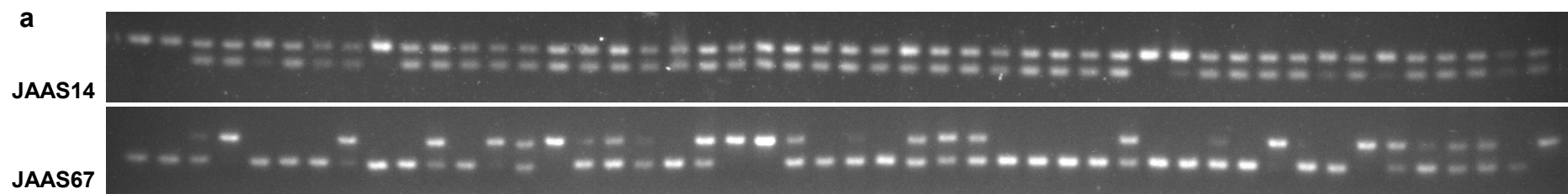

3% AGE

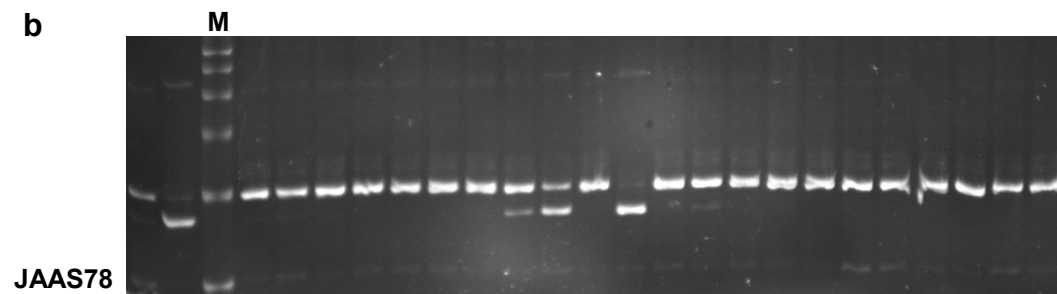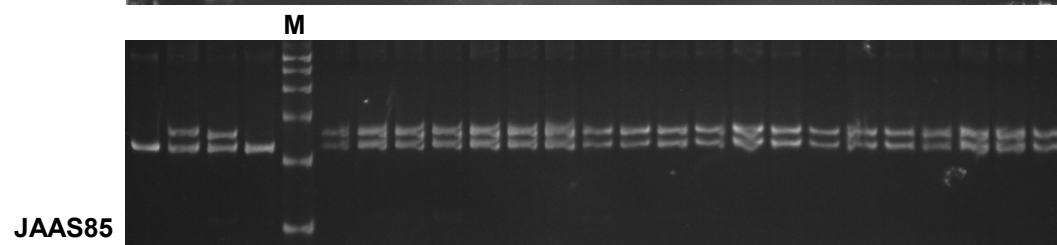

12% PAGE

Supplement: Supplementary file 5 — Fig. S1. Validation of predicted dimorphic InDel marker. Each lane represents one quinoa genotype. Homozygous genotype was indicated as one amplification band, while heterozygous genotype was indicated as two amplification bands per lane. a Validation of a marker with a large PCR length difference by 3% AGE without DNA marker (48 lanes). Markers JAAS14 and JAAS67 were 56 bp and 100 bp, respectively, as predicted from PCR length differences. b Validation of a marker with a small PCR length difference by 12% PAGE with DNA marker (25 lanes). The predicted PCR lengths of marker JAAS78 are 200 bp and 166 bp, while that of JAAS85 are 231 bp and 209 bp. M represents DNA marker I with six DNA fragment sizes (600 bp, 500 bp, 400 bp, 300 bp, 200 bp, and 100 bp). (PDF 687 kb) [file 12864_2017_4093_MOESM5_ESM.pdf]

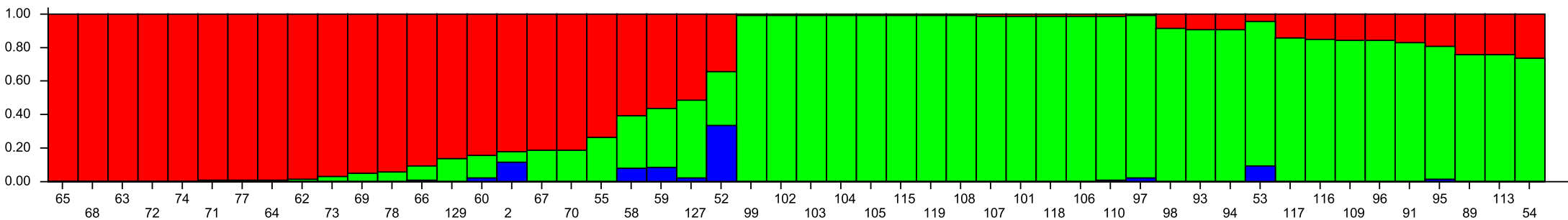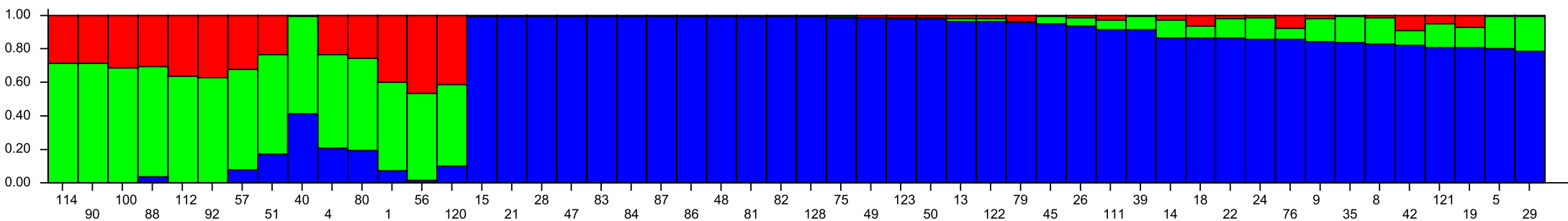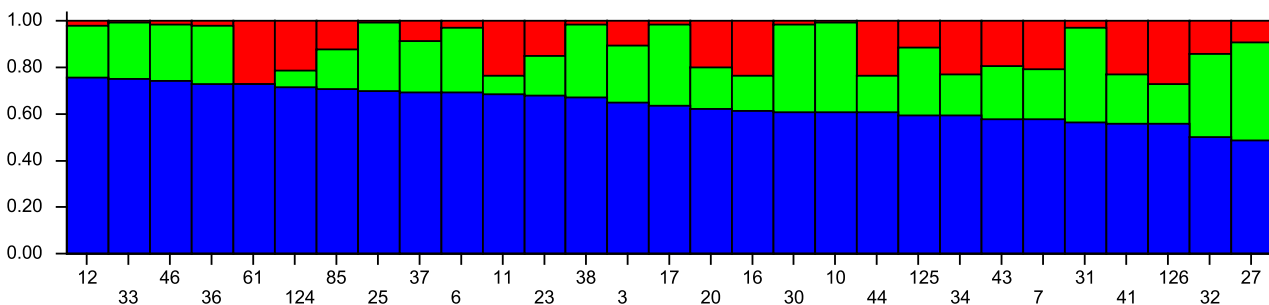

Supplement: Supplementary file 7 — Fig. S2. Population structure of the 129 quinoa accessions shown via STRUCTURE (K = 3). Each quinoa accession is represented by a vertical bar. Numbers on the y-axis indicate the membership coefficient, while the numbers on the x-axis indicate the serial number of each accession. The three groups are marked separately by red, green and blue colors. (PDF 104 kb) [file 12864_2017_4093_MOESM7_ESM.pdf]

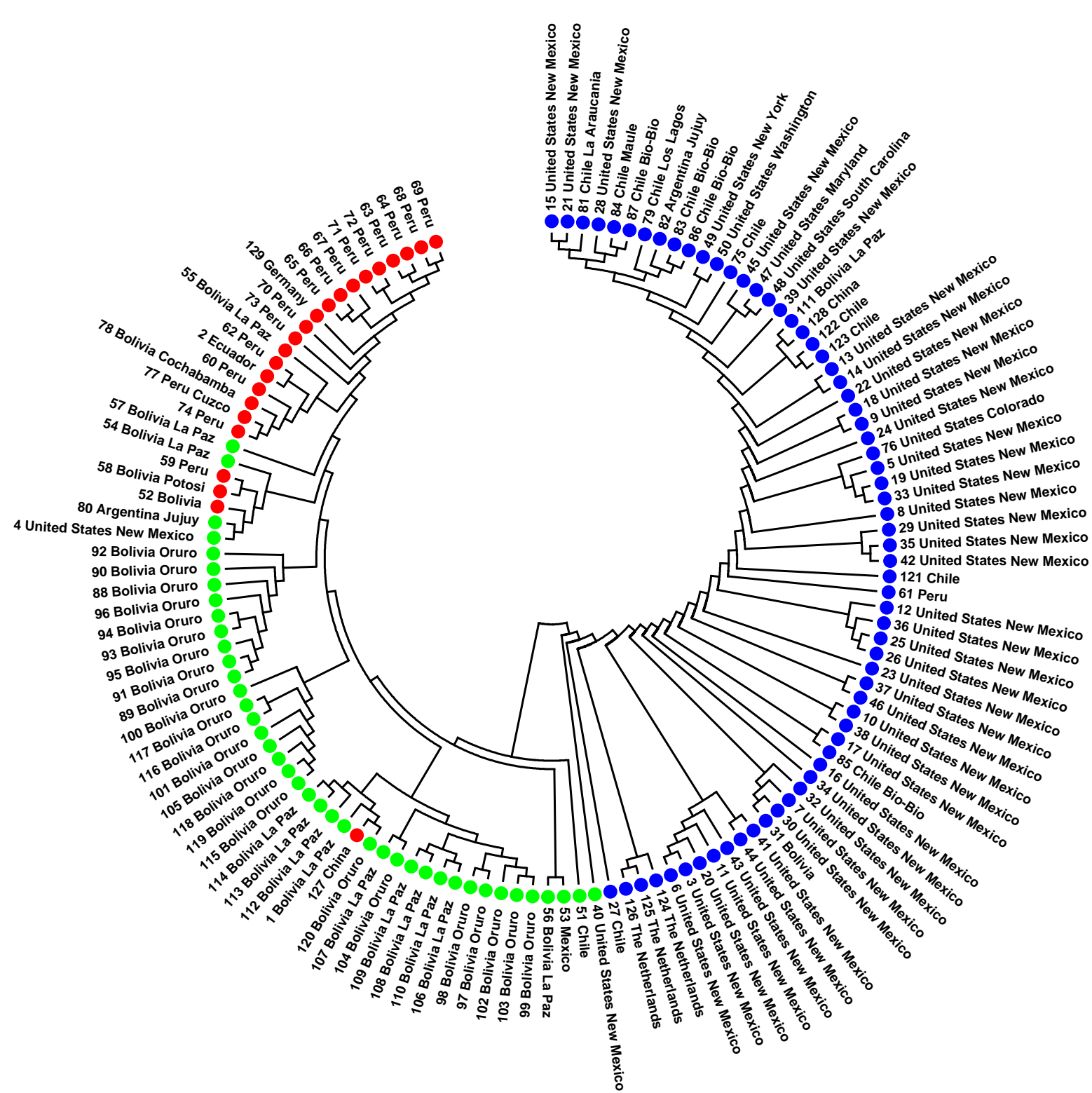

Supplement: Supplementary file 8 — Fig. S3. The NJ tree of the 129 quinoa accessions based on Nei’s genetic distance (1983) calculated from the 147 markers. The quinoa accessions are color-coded based on the groups identified by STRUCTURE (K = 3). (PDF 163 kb) [file 12864_2017_4093_MOESM8_ESM.pdf]
